# Dynamic Alteration of Microbial Communities of Duckweeds from Nature to Nutrient-Deficient Condition

**DOI:** 10.3390/plants11212915

**Published:** 2022-10-29

**Authors:** Chakrit Bunyoo, Peerapat Roongsattham, Sirikorn Khumwan, Juthaporn Phonmakham, Passorn Wonnapinij, Arinthip Thamchaipenet

**Affiliations:** 1 Interdisciplinary Graduate Program in Bioscience, Faculty of Science, Kasetsart University, Bangkok 10900, Thailand; 2 Department of Genetics, Faculty of Science, Kasetsart University, Bangkok 10900, Thailand; 3 Duckweed Holobiont Resource & Research Center (DHbRC), Kasetsart University, Bangkok 10900, Thailand; 4 Omics Center for Agriculture, Bioresource, Food and Health Kasetsart University (OmiKU), Bangkok 10900, Thailand

**Keywords:** duckweed, microbiome, 16S rRNA, metagenome, stress

## Abstract

Duckweeds live with complex assemblages of microbes as holobionts that play an important role in duckweed growth and phytoremediation ability. In this study, the structure and diversity of duckweed-associated bacteria (DAB) among four duckweed subtypes under natural and nutrient-deficient conditions were investigated using V3-V4 16S rRNA amplicon sequencing. High throughput sequencing analysis indicated that phylum Proteobacteria was predominant in across duckweed samples. A total of 24 microbial genera were identified as a core microbiome that presented in high abundance with consistent proportions across all duckweed subtypes. The most abundant microbes belonged to the genus *Rhodobacter*, followed by other common DAB, including *Acinetobacter*, *Allorhizobium-Neorhizobium-Pararhizobium-Rhizobium*, and *Pseudomonas*. After nutrient-deficient stress, diversity of microbial communities was significantly deceased. However, the relative abundance of *Allorhizobium-Neorhizobium-Pararhizobium-Rhizobium*, *Pelomonas*, *Roseateles* and *Novosphingobium* were significantly enhanced in stressed duckweeds. Functional prediction of the metagenome data displayed the relative abundance of essential pathways involved in DAB colonization, such as bacterial motility and biofilm formation, as well as biodegradable ability, such as benzoate degradation and nitrogen metabolism, were significantly enriched under stress condition. The findings improve the understanding of the complexity of duckweed microbiomes and facilitate the establishment of a stable microbiome used for co-cultivation with duckweeds for enhancement of biomass and phytoremediation under environmental stress.

## 1. Introduction

Duckweeds, tiny flowering aquatic plants, belong to the family *Lemnaceae*, consisting of five genera; *Spirodela*, *Landoltia*, *Lemna*, *Wolffia*, and *Wolffiella*. Presently, 36 species of duckweed have been identified worldwide [1]. Duckweeds have been intensively studied in terms of aquatic plant models, animal feed, human food, biofuel production, and wastewater treatment due to their richness of nutrition, as well as their capability for phytoremediation [2,3,4,5,6].

Recently, several potential plant-growth-promoting bacteria (PGPB) have been isolated from duckweeds [7,8]. For instance, *Acinetobacter calcoaceticus* P23 has been proven to benefit *Lemna aoukikusa* by promoting biomass and facilitating phytoremediation [7,9]. Co-cultivation of two PGPB strains, *Ac. calcoaceticus* P23 and *Pseudomonas* sp. Ps6, enhanced growth of *Lemna minor* [10]. An indigenous wastewater bacteria, *Chryseobacterium* sp. 27AL, promoted biomass production of *Lemna gibba* under N-rich wastewater and limited-N conditions [11]. Using next-generation sequencing, the microbial community associated with natural growing duckweeds has been identified to consist of members in phyla Proteobacteria, Bacteroidetes, Firmicutes, and Actinobacteria [12]. However, the application of those PGPB was limited by the competitive indigenous community. For example, inoculation of *Aquitalea magnusonii* H3 to *L. minor* promoted duckweed biomass at the beginning of the association, but gradually lost its benefit due to the indigenous community competition [13]. Moreover, the duckweed-inoculated microbial community was changed over time during phytoremediation process [5]. Environmental abiotic factors, such as salinity, also altered the bacterial community of *L. minor* [14]. Thus, a successful PGPB inoculant should be able to invade and persist against both indigenous bacteria and variable abiotic stresses. Understanding the interaction between duckweeds and PGPB towards a dynamic change of the associated microbes over various environmental conditions is necessary to improve PGPB application [15].

In this work, the duckweed-associated bacterial community of four subtypes of natural growing duckweeds, *Spirodela*, *Landoltia*, *Lemna*, and *Wolffia*, in Thailand, were investigated using a metagenomic approach. Microbiome profiles of natural duckweeds before and after growing in an extreme nutrient-deficient condition were examined, and a set of “core” microbiomes were identified. The findings in this study will enhance the understanding of duckweed-microbial communities for the establishment of a stable PGPB community used for duckweed applications. 

## 2. Results

### 2.1. Microbial Diversity and Composition

Duckweeds in natural conditions (NC) were identified as *Landoltia punctata*, *Lemna aequinoctialis*, *Spirodela polyrhiza*, and *Wolffia globose*, based on two-barcode approaches (data not shown). Chemical composition of ambient (surrounding) water (AW) composed of 35.53 mg/L of total N and 0.71 mg/L of NO_3_-N, at pH 7.53 with 0.227 dS/m of EC. No P, K, As, or Cd were detected. To evaluate the dynamic change of duckweed microbial communities under nutrient-deficient conditions (stress condition; SC), NC duckweeds were grown in sterilized distilled water at 25 °C under a 12-h photoperiod corresponding to the ambient temperature and daylight hours of NC. This condition was adopted to minimize the effect of environmental conditions that may alter bacterial communities. After cultivation under SC for two weeks, the growth of the NC duckweeds was retarded, and half of them turned yellowish and pale (data not shown). 

Microbiomes of NC (*n* = 20) and SC (*n* = 20) duckweeds, as well as AW (*n* = 5), were determined using V3–V4 region of 16S rRNA gene amplicon sequencing. Chimera, chloroplast, mitochondria, and low frequency ASVs were removed from the total 5,932,394 reads of 50 data sets to obtain 3,123,655 reads (Table S1). The number of processed reads per sample ranged between 27,601 to 95,416 with a median of 59,800 (Table S2). To minimize bias introduced by the magnitude of sample depth, all samples were rarefied to an even number of 27,601 reads prior to diversity analysis (Figure S2). The number of detected ASVs and the calculated diversity index across samples are listed in Table S3. The highest number of ASVs was detected in NC *Spirodela* (1,345 ASVs), while the lowest number was that of SC *Wolffia* (449 ASVs) (Table S3). 

**Figure 1:**
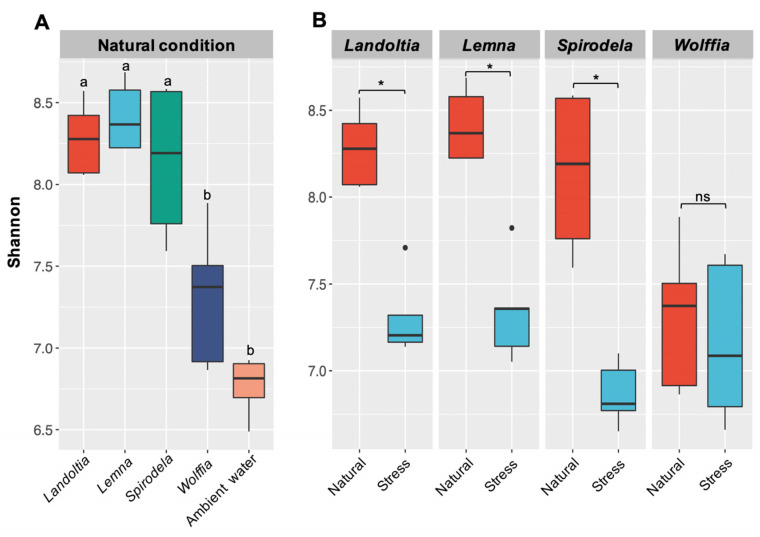
Comparison of alpha diversity index (Shannon). (**A**) Between natural duckweed subtypes and ambient water; and (**B**) between natural and nutrient-deficient (stress) conditions. Asterisks (*) indicate significant difference based on Kruskal-Wallis test, *p*-value < 0.05; ns, no significant difference.

Within sample diversity (alpha diversity) based on the Shannon index, NC *Landoltia*, *Lemna*, and *Spirodela* harbored microbial diversity higher than that of AW (Figure 1A; *p*-vaule < 0.05); whereas NC *Wolffia* revealed the smallest degree of microbial diversity across all duckweeds. NC duckweeds harbored microbial communities with a similar degree of diversity, with a Shannon index ranging from 7.59–8.57 for *Landoltia*, *Lemna*, and *Spirodela*, and except *Wolffia*, which displayed a significantly smaller Shannon index (6.92–7.50) (Table S3). There was no significant difference observed in the Shannon index between NC *Wolffia* and AW (Figure 1A; *p*-value = 0.07). After a 2-week nutrient starvation, most of the duckweeds, except *Wolffia*, loosened their microbial diversity (Figure 1B). However, the reduction of bacterial diversity was observed in SC *Wolffia* without statistical significance (Figure 1B; *p*-value = 0.67). 

**Figure 2:**
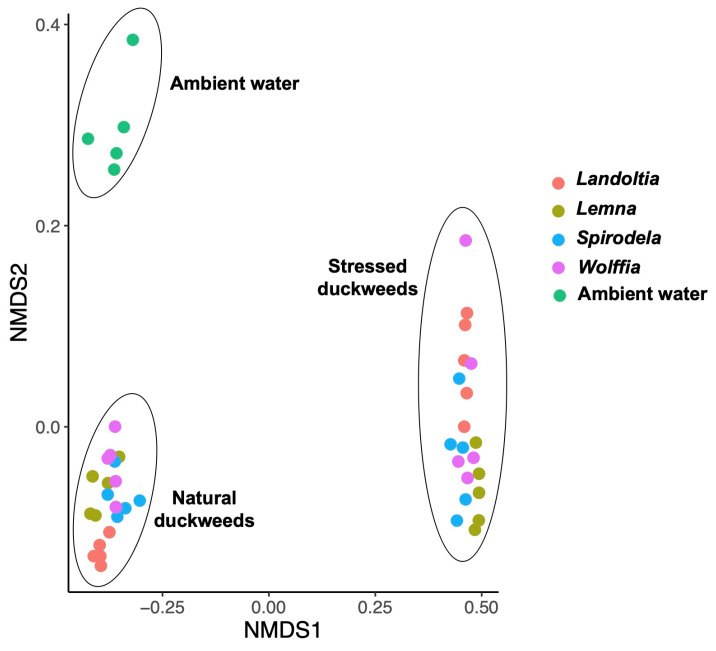
Non-metric multidimensional scaling (nMDS) analysis of the Bray-Curtis dissimilarity index between natural, stressed duckweeds (nutrient deficiency) and ambient water. All conditions displayed significant difference based on PERMANOVA analysis (*p*-value < 0.01).

Diversity between samples (beta diversity) based on the Bray-Curtis dissimilarity matrix was calculated to estimate the effect of environmental conditions and duckweed subtypes in shaping the microbiome community. The microbial communities associated with the same environmental conditions were likely to be clustered. Conversely, the microbial communities from different environmental conditions were clearly distinguished on the nMDS ordination (Figure 2). PERMANOVA analysis strongly supported the nMDS ordination result. The microbial communities detected in NC, SC, and AW were significantly different from one another (*p*-value < 0.001). 

**Figure 3:**
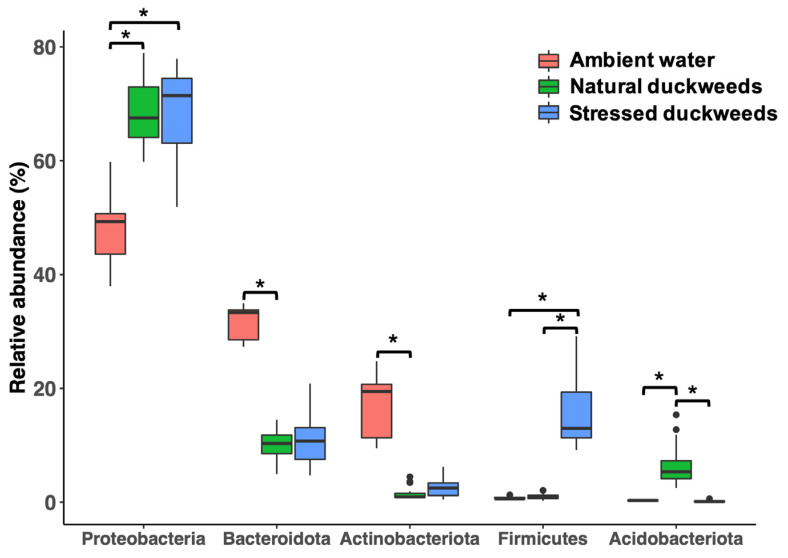
Taxonomic composition of microbial community associated with natural, stressed duckweeds (nutrient deficiency) and ambient water. The most abundance phyla (>5% median relative abundance) were plotted. Asterisks (*) indicate significant difference based on Wilcoxon test, *p*-value < 0.05. Dots indicate potential outliers.

Microbial taxa composition of NC and SC duckweeds, as well as AW, were classified into taxonomic levels. Proteobacteria was the most prominent phylum across samples (67.5%, 71.4%, and 49.3% median relative abundance of NC, SC, and AW, respectively; Figure 3). For NC duckweeds, the highly abundant phyla consisted of Bacteroidota (10.3%) and Acidobacteriota (5.3%). Whereas, Firmicutes, Bacteroidota, and Actinobacteria were prevalent in SC duckweeds with median relative abundances of 12.9%, 10.7%, and 2.4%, respectively (Figure 3). Conversely, AW harbored Bacteroidota and Actinobacteria with median relative abundances of 33.3% and 19.4%, respectively (Figure 3). Although Proteobacteria were predominant throughout the samples, the median relative abundance of Proteobacteria in AW was significantly lower than those of NC and SC duckweeds (Figure 3). However, Bacteroidota and Actinobacteria detected in AW displayed a median relative abundance greater than that of NC and SC duckweeds. Furthermore, Firmicutes were detected in SC duckweed with a median relative abundance significantly higher than that of NC duckweed and AW (Figure 3). 

### 2.2. Core Microbiomes of Natural Duckweed

**Figure 4:**
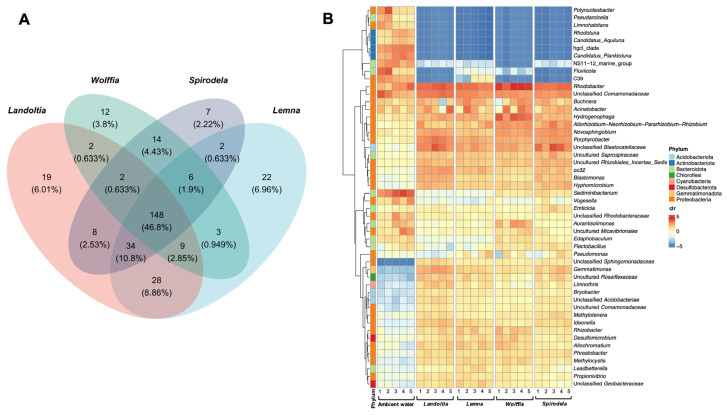
Candidate core microbiomes defined by bacterial genera that presented in every biological replicate of duckweed subtypes and ambient water, disregarding their relative abundance using (**A**) Venn diagram, and (**B**) heatmap representing the top 50 abundant core genera.

To evaluate the core bacterial community associated with NC duckweeds, all ASVs were analyzed at the genus level. A sum of 315 putative core microbiomes were defined by the bacterial genera that presented in every biological replicate of each duckweed subtype, disregarding their relative abundances (Table S4). Almost half of overall genus candidates (148 of 315; 46.8%) were consistently found among the four duckweed species (Figure 4A). There were bacterial genera that were exclusively detected in *Landoltia*, *Lemna*, *Spirodela*, and *Wolffia* at 6.0% (19 of 315), 6.9% (22 of 315), 3.8% (7 of 315), and 8.8% (12 of 315), respectively (Figure 4A). However, these unique genera presented rather low relative abundances ranging between 0.01% to 0.60%; whereas those of conserved core genera varied between 0.01% to 20.2% across the four duckweed species. In order to define low or high abundance taxa, the counts or taxa abundances were transformed into centered log ratio (clr) where the abundance counts were compared to their geometric mean. The taxa carrying clr values close to 0 indicated their average abundance. By this criterion, the majority of the conserved genera (141 of 148; 95.2%) displayed abundance above average (clr > 0; Table S5), while all unique genera showed low abundance (clr < 0; Table S5). The results indicated that the moderate to high abundances of microbial community were shared across duckweed subtypes.

*Rhodobacter* was predominant in almost duckweed subtypes, including *Landoltia*, *Lemna*, and *Wolffia*, with average relative abundances of 8.9%, 8.9%, and 20.2%, respectively; whereas the unclassified genus of the family *Blastocatellaceae* was prominent in *Spirodela,* followed by *Rhodobacter*, with average relative abundances of 9.4% and 9.2%, respectively (Table S6). There were 24 genera presented in high abundance (>1% relative abundance; clr > 3) with consistent proportion across all duckweed subtypes (Table S5). The most abundant were *Acinetobacter*, *Allorhizobium*-*Neorhizobium*-*Pararhizobium*-*Rhizobium*, *Hydrogenophaga*, *Novosphingobium*, *Porphyrobacter*, and *Rhodobacter* (Figure 4B). For ambient water, a total of 156 genera were defined as putative core microbiomes of which *Sediminibacterium* was predominant (12.8% relative abundance), followed by hgcl clade, an unclassified genus of the families *Comamonadaceae*, *Fluviicola*, and *Rhodobacter*, with relative abundances of 6.8%, 6.8%, 6.3%, and 5.7%, respectively (Table S5). Of those, *Rhodobacter*, an unclassified genus of the families *Comamonadaceae*, *Buchnera*, and *Acinetobacter*, were also found in NC and SC duckweeds (Figure 4B). 

### 2.3. Nutrient-Deficient Condition Altered Duckweed Core Microbiomes

After treating four subtypes of NC duckweeds under stress of nutrient starvation, 174 bacterial genera were observed as putative core microbiomes. Approximately, 20% of putative core genera (38 of 174) were conserved across all duckweed subtypes (Figure S3). Members of the putative core genera identified in SC duckweeds were less than those of NC duckweeds (174 vs. 315 genera). The results indicated that most duckweeds lost their core microbial community under nutrient-deficient conditions, which was clearly supported by alpha diversity analysis (Figure 1B). 

The most abundant core microbiome exhibited in *Landoltia* was a member of genus *Allorhizobium-Neorhizobium-Pararhizobium-Rhizobium*, with a relative abundance of 17.8%, followed by unclassified genera of the families *Comamonadaceae*, *Pelomonas*, *Roseateles*, and *Novosphingobium*, with relative abundances of 13.3%, 12.7%, 10%, 10.6%, and 6.4%, respectively (Table S7). Similarly, *Allorhizobium-Neorhizobium-Pararhizobium-Rhizobium* was the most frequent core genus found in *Wolffia*, with a relative abundance of 14.5%, followed by *Pelomonas*, *Novosphingobium*, *Roseateles*, and an unclassified genus of the family *Comamonadaceae*, with relative abundances of 12.3%, 8.2%, 7.9%, and 7.8%, respectively (Table S7). *Roseateles* was the predominant core genus detected in *Lemna*, with a relative abundance of 12.4%, followed by *Allorhizobium-Neorhizobium-Pararhizobium-Rhizobium*, *Novosphingobium*, *Pelomonas*, and *Curvibacter,* with relative abundances of 11.1%, 6.8%, 5.3%, and 4.7%, respectively (Table S7). *Spirodela* harbored a majority of *Allorhizobium-Neorhizobium-Pararhizobium-Rhizobium*, with a proportion of 14.9%, followed by unclassified genera of the families *Comamonadaceae*, *Roseateles*, *Novosphingobium*, and *Pelomonas*, with proportions of 14.7%, 8.5%, 8.4%, and 7.1%, respectively (Table S7). 

**Figure 5:**
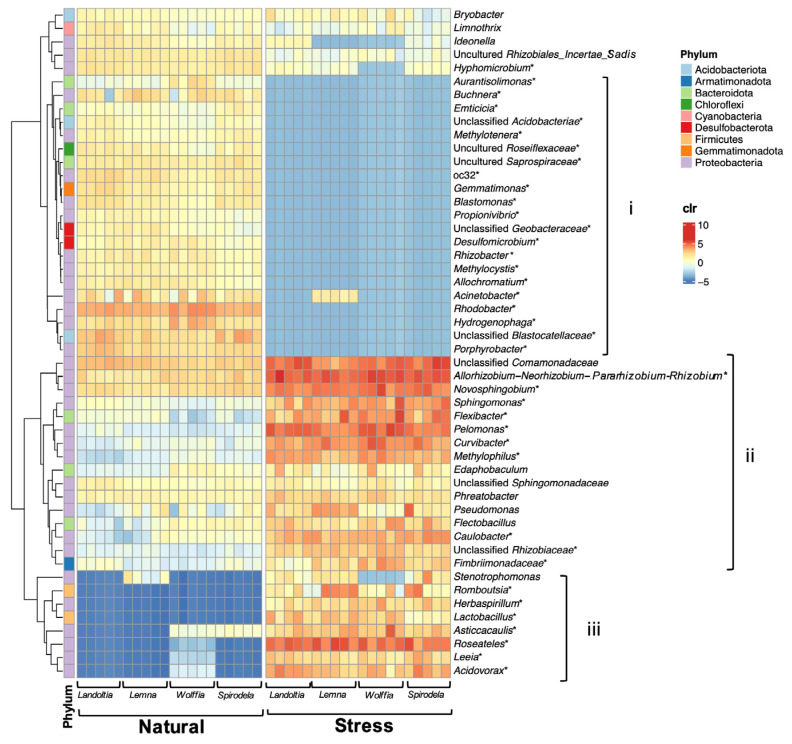
Top 50 abundant core genera of natural and stressed duckweeds (nutrient deficiency). The alteration of core microbiomes was categorized into: (**i**) high abundance in natural duckweeds but diminished under stress; (**ii**) high abundance in natural duckweeds and enriched under stress; and (**iii**) low abundance in natural duckweeds but highly enriched under stress. Asterisks (*) indicate significant difference based on ALDEx2, *p*-value < 0.05.

Differential abundance of core microbial communities between NC and SC duckweeds displayed around 34% of the core genera (147 of 427) with significant differences (Table S8). Under nutrient-deficient stress, the core microbiomes were dynamically changed (Figure 5). Of those, the prominent genera consistently found in NC duckweeds, such as *Rhodobacter* and *Acinetobacter*, were significantly diminished under nutrient-deficient stress (Figure 5, Table S8). Interestingly, rare core microbiomes in the phylum *Proteobacteria*, including *Roseateles*, *Sphingomonas*, and *Pelomonas*, detected in NC duckweeds were greatly enhanced under SC treatments, followed by members in the phylum *Firmicutes*, such as *Lactobacillus* and *Romboutsia* (Figure 5, Table S8). In addition, the genus *Allorhizobium-Neorhizobium-Pararhizobium-Rhizobium*, which presented with high abundance in NC duckweeds, were increased under SC treatments. 

### 2.4. Functional Prediction of Microbial Communities of Duckweeds

**Figure 6:**
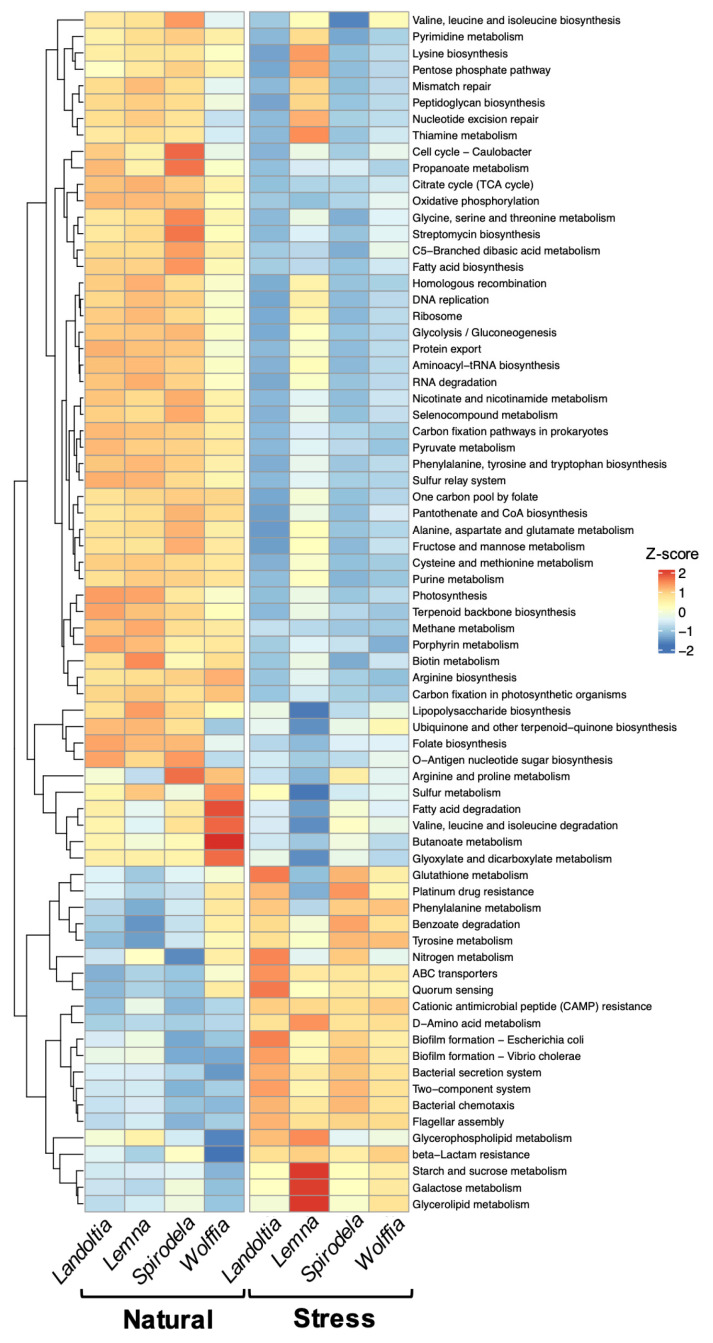
Functional prediction of bacterial communities of duckweeds predicted by PICRUSt2. Relative abundance of pathways that are significantly different (*p*-value < 0.05) between natural and stressed duckweeds are shown.

Based on KEGG orthologs, 84 functional pathways of duckweed microbial communities were predicted (Table S9). Of those, 73 pathways were categorized into metabolism, environmental information processing, cellular processes, genetic information processing, and drug resistance that were significantly different between NC and SC duckweed microbial communities (Figure 6). Under stress conditions, the relative abundance of four pathways in cellular processes were significantly enriched, including bacterial chemotaxis, biofilm formation, flagellar assembly, and quorum sensing. The pathways involved in environmental information processing also displayed relative abundance enrichment, such as ABC transporters, bacterial secretion systems, and two-component systems. Furthermore, relative abundance of nitrogen metabolism related to plant growth, promoting function and benzoate degradation involved in biodegradation, were significantly increased (Figure 6). Conversely, the pathways mainly related to amino acid metabolism displayed significantly lower relative abundance in SC duckweed microbial communities (Figure 6). 

## 3. Discussion

Duckweeds are known to be associated with beneficial PGPB as holobionts [8]. These associated bacteria help promote duckweed growth and phytoremediation performance [7,9]. However, utilization of these PGPB in the real environment is limited, since understanding of the interaction between host and microbes in various environmental conditions is required [15]. Here, we investigated a duckweed-associated microbial community which has not yet been reported in this region using 16S rRNA amplicon metagenome strategy. The “core” microbiomes of the four subtypes of duckweeds, both in natural and stress conditions, were identified. 

The natural condition (NC) duckweeds from the same location harbored microbial communities with similar degrees of diversity, except *Wolffia*. This may suggest that the rootless morphology of *Wolffia* and its physiology affected the microbial diversity. The microbial richness (observed ASVs) of NC duckweeds in this study, ranging from 769–1345 ASVs (Table S3), was greater than those of duckweeds collected from ponds in the U.S. [12]. The divergence of the microbial diversity among different studies was possibly caused by the distinction of geographic locations, environmental conditions or variation of methodologies in those studies, such as sample collection approaches, DNA preparation protocols, sequencing depths, and 16S rRNA regions.

The microbial diversity of NC, SC duckweeds, and the ambient water (AW) were clearly distinguished. The bacterial communities of most subtypes of NC duckweeds in this study were higher than those of AW, which agrees with the richness of bacterial diversity of duckweeds collected in Japan that was higher than that in their surrounding water [16]. Conversely, the microbial diversity of duckweeds collected in the U.S. was lower than in their surrounding water [12]. These findings indicate that the floating duckweeds directly interacted with water act as a microbial shelter in aquatic environments, and the associated bacterial communities under the same environmental conditions are likely to be clustered. The different sources of water influenced the microbial assemblage of duckweeds that changed the microbial communities [17]. The results of this study also suggested that environment conditions have a forceful consequence on duckweed microbial composition rather than the duckweed subtypes. In land plants, soil components were an important factor that significantly influenced the soil microbiome [18,19]. However, further investigation is required to determine the chemical composition of water that possibly affects the duckweed bacterial assembly. When the four subtypes of NC duckweeds were cultivated in nutrient-deficient environments, most of the duckweeds significantly lost their bacterial diversity. The results agree with the diversity of *L. minor* microbiome, which was reduced under salinity stress [14]. 

Microbiomes of NC and SC duckweeds, and of AW, displayed Proteobacteria as the most predominant phylum, similar to results outlined in previous reports [12,16,20]. Members of Proteobacteria, followed by Bacteroidota, were the major phyla found in NC duckweeds, which was supported by previous duckweed microbiome studies [12,16]. In general, Proteobacteria and Firmicutes were dominant phyla in plant endospheres, while Proteobacteria and Bacteroidota were mainly composed in phyllospheres [21]. Pangenomic analysis of Proteobacteria isolated from roots of *Brassicaceae*, poplar, and maize revealed a higher number of substrate transporters that could export/import a board range of compounds [22]. This may explain their high abundance in plant environments and may enable fast-growing characteristics during nutrient-deficient conditions [21]. Moreover, the proportion of Firmicutes was significantly enhanced in SC duckweeds compared to that of NC. The Firmicutes were previously enriched during a period of drought stress due to their thicker cell walls, which promoted their stress tolerance [23,24]. However, most plant-microbe interaction studies have been performed on terrestrial plants, and so the nature of duckweed holobionts in an aquatic lifestyle remains unclear.

To shade more light on duckweed-associated microbes, the microbial community profiles were classified at the genus level. The term “core” microbiome aims to identify a group of potential microbes that are consistently present in duckweed hosts [12]. In this study, half of the core microbiomes were shared across the four subtypes of duckweed collected from the same natural site. The data suggested that duckweeds growing in the same location harbored a remarkable conserved core microbiome across duckweed subtypes. Core genera exclusively found in one subtype but not in the others, presented in small relative abundance (0.01–0.60%), which did not represent specific taxa, but likely occurred due to non-captured sequencing. 

Several dominant genera of Proteobacteria, such as *Acinetobacter*, *Allorhizobium*-*Neorhizobium*-*Pararhizobium*-*Rhizobium*, *Hydrogenophaga*, *Novosphingobium*, and *Rhodobacter*, were found to be universal “core” microbiomes of NC duckweeds, which were similarly observed as duckweed microbiomes [12,16]. These stable genera suggested that the core microbiomes tentatively act as the universal duckweed-associated bacteria (DAB). *Acinetobacter calcoaceticus* P23 has been proven to promote duckweed biomass and facilitate phytoremediation through phenol degradation [7,9]. Members of the genus *Rhizobium* are well-known as a typical symbiosis of leguminous plants, as well as the other plants [25]. Several species of *Novosphingobium* promoted plant growth by the production of indole-3-acetic acid (IAA) [26,27,28]; while members of *Rhodobacter* were recognized as plant growth-promoting bacteria [29,30]. Although many potential DAB have been successfully inocculated to duckweeds through culture-dependent [7,8,16] and culture-independent methods [12,16], associations occurred for a short period and then vanished [13]. Therefore, high potential DAB suitable for long-term applications should be selected from “stable” core microbiomes. In addition, some common DAB found in NC duckweeds, such as *Rhodobacter* and *Acinetobacter*, are also present in ambient water. The results suggest that those core genera, originally exhibited in the surrounding water, were exclusively recruited by the duckweed host [12,17]. 

The “stable” core microbiomes of four subtypes of duckweeds under nutrient-deficient conditions were investigated. The candidate-beneficial DAB, such as *Acinetobacter* and *Rhodobacter*, found highly abundantly in NC duckweed, were significantly diminished; whereas *Allorhizobium*-*Neorhizobium*-*Pararhizobium*-*Rhizobium* and *Novosphingobium* were persistently enhanced. The results suggested that these potential duckweed-associated microbiomes experience a dynamic change in response to the environment or stressor. The disappearing scenario was observed when a beneficial DAB, *Aquitalea magnusonii* H3, was inoculated to *L. minor*; it could promote growth in just a week and vanished after growing under several conditions [13]. Members of the genus *Rhizobium* could promote drought tolerance in both leguminous [31,32] and non-leguminous plants [33]. Genome analysis of *Rhizobium* strains revealed a set of genes that are involved in plant-growth-promoting and stress-tolerant traits, including phosphate solubilization, production of IAA, exopolysaccharide, siderophores, and 1-aminocyclopropane-1-carboxylate (ACC) deaminase [32]. A *Novosphingobium* strain was reported to increase salinity tolerance and induce accumulation of IAA in plants [34]. Interestingly, several rare core microbiomes belonged to the phylum Proteobacteria, such as *Pelomonas*, *Roseateles*, and *Sphingomonas*, and phylum Firmicutes, such as *Lactobacillus*, were also detected in NC duckweeds and were greatly enhanced under nutrient deficiency. These genera have been reported as PGPB; for instance, *Pelomonas* sp. MRB3 has been recently proven as a DAB by root colonization and growth promotion of *L. minor* [35]. An endophytic *Sphigomonas* was reported to promote growth of tomatoes by the production of phytohormones, IAA, and gibberellins [26]. Genome analysis of *Sphingomonas*-determined genes related to adaptation to extreme oligotrophic environments [36]. Similarly, *Lactobacillus*, associated with plants, displayed plant growth promoting traits, such as IAA production, phosphate solubilization, and anti-phytopathogenic activity [37,38] which could be applied as biofertilizer in a variety of plants, such as wheat, tomato, pepper, and cucumber [37]. Apart from PGP traits, *Roseateles depolymerans* TB-87, isolated from fresh water, was reported to be able to decompose various bioplastics that may be useful for bioremediation [39]. 

Functional predictions of microbial communities in NC and SC duckweeds displayed alterations in the relative abundance of the pathways. Under stress conditions, pathways involved in bacterial motility, biofilm formation, chemotaxis, flagellar assembly, and two-component systems were significantly enhanced. The findings are comparable to those functional predictions of natural water-obtained DAB, co-cultivated with several duckweed species, including *Spirodela*, *Landoltia*, *Lemna*, *Wolffiella*, and *Wolffia* [13,17]. These enhanced pathways may explain essential steps for DAB colonization. A recent study on duckweed illuminated these functions, particularly flagellar motility and cell surface structures such as lipopolysaccharide and type-IV pili synthesis, were essential for colonization and fitness regulation of DAB, *A. magnusonii* H3, to *L. minor* surfaces [40]. Comparable to those of terrestrial plants, *Arabidopsis thaliana* attracted beneficial *Bacillus subtilis* via root exudates, while flagellar motility and chemotaxis machinery mediated *B. subtilis* contact and settled on the roots before forming into biofilm for long-term colonization [41]. Two component system signal transduction is the key pathway involved in differentiation of bacterial cells to biofilm-producing cells [42]. Similarly, biofilm formation contributed to corn root colonization and seed adhesion of plant-beneficial *Pseudomonas putida* KT2440 [43,44]. Additionally, the relative abundance of benzoate degradation and nitrogen metabolism pathways are significantly increased in the microbial communities of SC duckweeds, which may suggest the enhancement of the bioremediation ability of those DAB, such as phenolic compound degradation and nitrogen removal [13,17]. The functional prediction results suggested that those persistently presented bacteria were likely to be a real DAB, and have a positive effect on duckweed fitness under stress conditions. 

Altogether, *Lactobacillus*, *Novosphingobium*, *Pelomonas*, *Rhizobium*, *Roseateles*, and *Sphingomonas* are proposed to be “stable” DAB of duckweeds; potential candidates for duckweed utilization under stress environments. Further investigation is required to understand the actual DAB traits and their interaction with the duckweed host.

## 4. Materials and Methods

### 4.1. Sample Collection

Four duckweed genera (*Spirodela*, *Landoltia*, *Lemna*, and *Wolffia*) and ambient (surrounding) water were collected from drainage ditches in Nakorn Pathom, Thailand (14°00′34.7″ N 99°58′13.3″ E) in June 2021 (Figure S1) in five replicates (duckweeds, *n* = 20; ambient water, *n* = 5). Duckweed samples were rinsed three times in sterilized water, transferred to 5 mL centrifuge tubes containing 3 mL of DNA/RNA shield^TM^ (Zymo Research Corp, Irvine, CA, USA), and immediately stored at −80 °C until used. An amount of 500 milliliters of water samples were passed through sterilized Whatman filter paper, grade 4 (20–25 µm), to get rid of impurities before being filtering through Whatman WME membrane (0.2 µm) to capture microbial communities. The filters were excised into small pieces and then transferred to 5 mL centrifuge tubes containing 3 mL of DNA/RNA shield^TM^ (Zymo Research Corp, Irvine, CA, USA) and immediately stored at −80 °C until used.

EC and chemical composition of the ambient water samples were analyzed for total N, P, K, As, Cd, and NO_3_-N at the Central Laboratory and Greenhouse Complex, Kasetsart University, Kamphaeng Saen Campus, Thailand. 

### 4.2. Experimental Design for Nutrient-Deficient Condition

Approximately 5 g fresh weight of natural duckweeds (*n* = 20) were grown in sterilized distilled water in clean glass containers (length × width × height: 31 × 18.5 × 19 cm) at 25 °C under a photo-period of 12 h with a light intensity of 50 µmol m^−2^ s^−1^. After 14-day cultivation, duckweed and water were harvested and preserved as described above. 

### 4.3. Duckweed Genotyping

Duckweed samples were identified by two barcodes: *atp*F-*atp*H (5′-ACTCGCACACACTCCCTTTCC-3′ and 5′-GCTTTTATGGAAGCTTTAACAAT-3′) and *psb*K-*psb*I (5′-TTAGCATTTGTTTGGCAAG-3′ and 5′-AAAGTTTGAGAGTAAGCAT-3′), using PCR conditions as previously described [45].

### 4.4. DNA Extraction and Sequencing

Approximately 250 mg of each duckweed sample obtained from natural and nutrient-deficient experiments were homogenized in liquid nitrogen. DNA from duckweed and water filtrates was isolated using a ZymoBIOMICS^TM^ DNA Miniprep Kit (Zymo Research Corp, USA) according to the manufacturer’s instructions. All DNA samples were quantified using a NanoDrop™ One/OneC Microvolume UV-Vis Spectrophotometer (Thermo Fisher Scientific, Waltham, MA, USA). Library was prepared using V3-V4 region of 16S rRNA amplification with primers 341F (5-CCTAYGGGRBGCASCAG-3) and 806R (5-GGACTACNNGGGTATCTAAT-3) [46]. Finally, 2 × 250 bp pair-end sequencing was performed using an Illumina Novaseq 6000 platform at NovogeneAIT Genomics Singapore Pte. ZymoBIOMICS^TM^ Microbial Community DNA Standard (Mock; Zymo Research Corp, CA, USA), and was used as a control.

### 4.5. Data Processing and Metagenome Analysis

The raw reads were pre-processed by the removal of adaptors and primers performed by NovogeneAIT. The pair-end reads were denoised, dereplicated, and chimeras-filtered using a dada2 plugin [47] under QIIME2 (q2) version 2021.8 [48]. The amplicon sequence variances (ASVs) were classified using a pre-formatted SILVA version 138 reference database [49] and q2-feature-classifier classify-sklearn. The suspected background contamination features were subtracted using Microdecon [50]. The final feature table was filtered for chloroplast, mitochondria, and low frequency ASVs (<10 reads across all samples).

Rarefaction analysis was conducted based on the feature table with a random sampling to the minimal read number (27,601) of all samples. Alpha diversity analysis was calculated using a q2-diversity alpha plugin. Non-parametric Kruskal-Wallis tests [51] were performed to compare the alpha diversity index. For beta diversity analysis, Bray-Curtis’s dissimilarity [52] was calculated using a q2-diversity core-metrics plugin. Permutation multivariate analysis of variance (PERMANOVA) [53] was used to compare community composition between groups of samples. Non-metric multidimensional scaling (nMDS) was calculated from Bray-Curtis’s dissimilarity matrix using the vegan package (https://CRAN.R-project.org/package=vegan (accessed on 24 February 2022)) and were plotted using the ggplot2 package [54] in R version 4.1.1. Differential abundance comparisons were performed using ALDEx2 [55]. The *p*-value obtained from multiple pairwise testing was adjusted by Holm’s sequential Bonferroni method [56]. Adjusted *p*-value < 0.05 was considered as statistically significant. Data visualization was conducted by ggplot2, ComplexHeatmap [57], and the VennDiagram package (https://CRAN.R-project.org/package=VennDiagram (accessed on 24 February 2022)) in R version 4.1.1.

### 4.6. Functional Metagenome Prediction

The obtained ASVs table was subjected to PICRUSt2 [58] to predict functional profiles of metagenome data. The predicted functional table was categorized into pathways based on the Kyoto Encyclopedia of Genes and Genomes (KEGG) Orthologs [59]. Those predicted pathways with low mean relative abundance (<0.4%) were filtered. The differential abundance of predicted pathways between natural and nutrient-deficient conditions was evaluated by the Wilcoxon (Mann-Whitney U) test [60]. 

## Data Availability

The 16S rRNA metagenome data were deposited in the GenBank NCBI Bioproject number PRJNA888649.

## Supplementary Materials

The following supporting information can be downloaded at: https://www.mdpi.com/article/10.3390/plants11212915/s1, Table S1 ASVs information and sequence reads for duckweeds under natural, nutrient-deficient conditions, and ambient water; Table S2 Number of sequences obtained from all samples; Table S3 Alpha diversity analysis of all samples with observed ASVs and Shannon diversity index; Table S4 Lists and abundance of core genera of duckweeds collected from natural site; Table S5 Lists of core microbiome obtained from four duckweed subtypes collected from natural site and ambient water; Table S6 High relative abundance (>1%) of core genera detected in duckweeds collected from natural site; Table S7 High relative abundance (>1%) of core genera detected in duckweeds under nutrient-deficient condition; Table S8 Differential abundance testing of bacterial communities between duckweeds in natural and in nutrient-deficient condition using ALDEx2; Table S9 Functional prediction of metagenome data using PICRUSt2; Figure S1, Duckweed samples collected from Kasetsart University Kamphaeng Saen campus, Nakhon Pathom, Thailand during June 2021; Figure S2, The rarefaction curve between observed ASVs and sample depth displayed nearly plateau at 27,601 reads; Figure S3, Candidate core microbiomes from duckweeds under nutrient-deficient condition.
