# Supplementary material for: Dynamic Alteration of Microbial Communities of Duckweeds from Nature to Nutrient-Deficient Condition
Source: Plants (Basel). 2022 Oct 29;11(21):2915. doi: 10.3390/plants11212915 (PMC9658847; doi:10.3390/plants11212915)
Supplement: Supplementary file 1 [file plants-11-02915-s001.zip › Bunyoo et al_Supplementary Figures.pdf]

Supplementary Figures

Dynamic alteration of microbial communities of duckweeds from nature to nutrient-deficient condition

Chakrit Bunyoo[1,2,3], Peerapat Roongsattham[2,3], Sirikorn Khumwan[2,3], Juthaporn Phonmakham[2,3], Passorn Wonnapinij[2,3,4], Arinthip Thamchaipenet[1,2,3,4,*]

[1]*Interdisciplinary Graduate Program in Bioscience, Faculty of Science, Kasetsart University, Bangkok 10900, Thailand*

[2]*Department of Genetics, Faculty of Science, Kasetsart University, Bangkok 10900, Thailand*

[3]*Duckweed Holobiont Resource & Research Center (DHbRC), Kasetsart University, Bangkok 10900, Thailand*

[4]*Omics Center for Agriculture, Bioresource, Food and Health, Kasetsart University (OmiKU), Bangkok 10900, Thailand*

* Correspondence, E-mail: arinthip.t@ku.ac.th

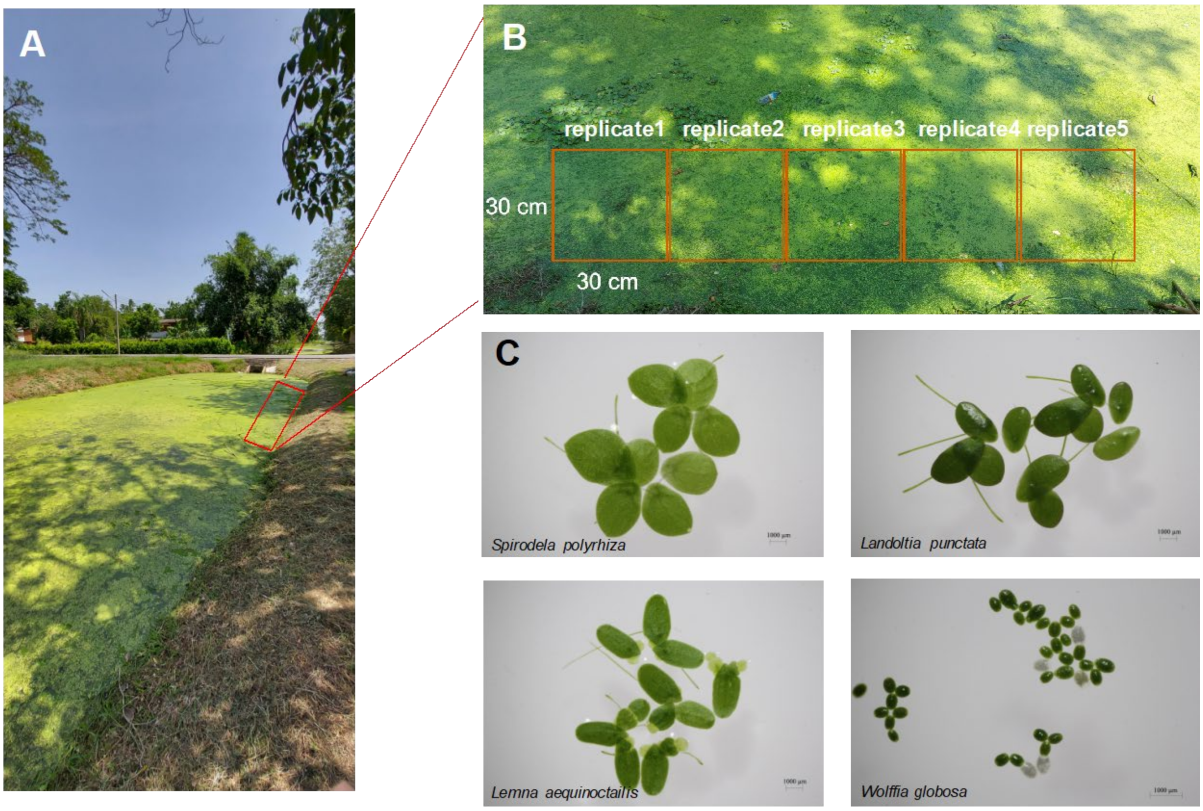

Figure S1 Duckweed samples collected from Kasetsart University Kamphaeng Saen campus, Nakhon Pathom, Thailand during June 2021. (A) Natural duckweeds in drainage ditch. (B) Collecting areas of five biological replicates of duckweed including ambient water. (C) Four species of duckweed identified as *Spirodela polyrhiza*, *Landoltia punctata*, *Lemna aequinoctialis* and *Wolffia globosa*.

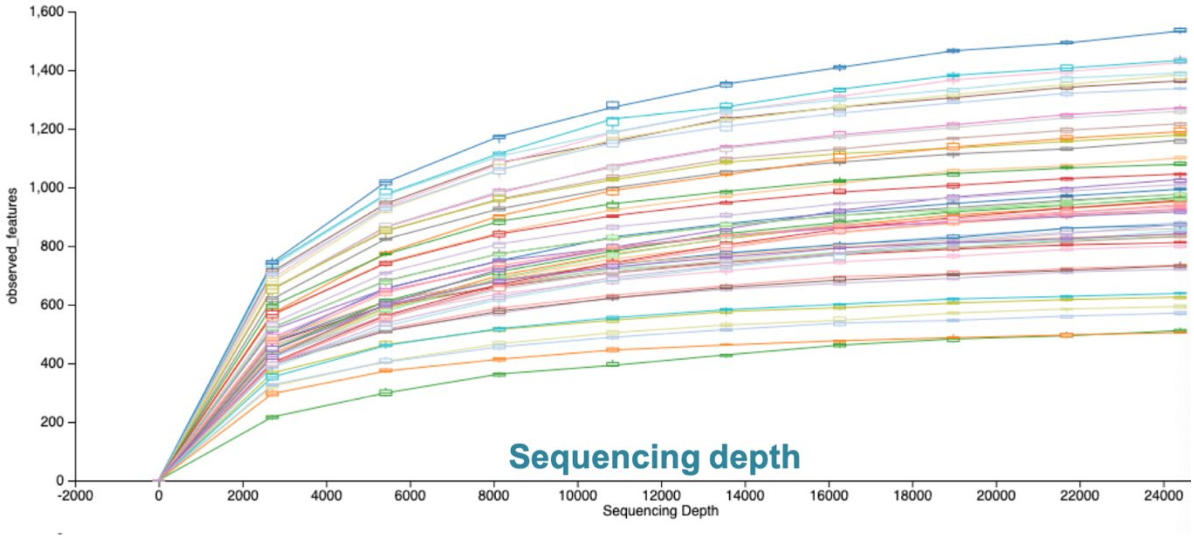

Figure S2 The rarefaction curve between observed ASVs and sample depth displayed nearly plateau at 27,601 reads.

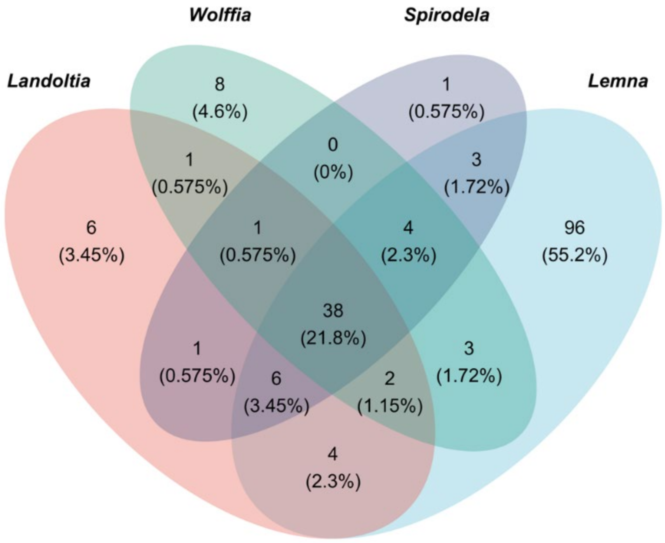

Figure S3 Candidate core microbiomes from duckweeds under nutrient-deficient condition.
